# The Denoising Method for Transformer Partial Discharge Based on the Whale VMD Algorithm Combined with Adaptive Filtering and Wavelet Thresholding

**DOI:** 10.3390/s23198085

**Published:** 2023-09-26

**Authors:** Zhongdong Wu, Zhuo Zhang, Li Zheng, Tianfeng Yan, Chunyang Tang

**Affiliations:** 1 School of Electronic and Information Engineering, Lanzhou Jiaotong University, Lanzhou 730070, China; 2 Silk Road Fantian (Gansu) Communication Technology Co., Ltd., Lanzhou 730070, China

**Keywords:** partial discharge, VMD, WOA, transformer, adaptive filtering, denoising

## Abstract

Partial discharge (PD) is the primary factor causing insulation degradation in transformers. However, the collected signals of partial discharge are often contaminated with significant noise. This makes it difficult to extract the PD signal and hinders subsequent signal analysis and processing. This paper proposes a denoising method for transformer partial discharge based on the Whale VMD algorithm combined with adaptive filtering and wavelet thresholding (WVNW). First, the WOA is used to optimize the important parameters of the VMD. The selected mode components from the VMD decomposition are then subjected to preliminary denoising based on the kurtosis criterion. The reconstructed signal is further denoised using the Adaptive Filter (NLMS) algorithm to remove narrowband interference noise. Finally, the residual white noise is eliminated using the Wavelet Thresholding algorithm. In simulation experiments and practical measurements, the proposed method is compared quantitatively with previous methods, VMD-WT, and EMD-WT, based on metrics such as SNR, RMSE, NCC, and NRR. The results indicate that the WVNW method effectively suppresses noise interference and restores the original PD signal waveform with high waveform similarity while preserving a significant amount of local discharge signal features.

## 1. Introduction

Power transformers have a significant impact on voltage transformation, distribution, and electrical energy transmission. They are essential components in power systems and are among the most crucial equipment [1]. Insulation faults are the main cause of transformer accidents, and the core reason behind transformer insulation faults is partial discharge (PD) [2]. Therefore, the detection of local discharge in transformers enables the analysis of the insulation condition, thereby achieving early warning and reducing operational failures. By detecting the physical phenomena generated by partial discharge, such as electrical pulses, ultrasonic waves, and chemical byproducts, the quantitative detection and localization of partial discharge can be achieved. Currently, methods used for PD signal detection can be classified into two categories based on the different physical and chemical processes: electrical measurement methods and non-electrical measurement methods [3]. Electrical measurement methods include the pulse current method, and Ultra High Frequency (UHF) detection method, etc. [4]. The pulse current method exhibits low measurement frequency, narrow bandwidth, and relatively limited information. Non-electrical measurement methods include ultrasonic testing [5], optical inspection methods, etc. [6]. The ultrasonic method has higher invasiveness and lacks sensitivity due to complex acoustic impedance. Optical inspection methods are sensitive to external light and environmental conditions, making them susceptible to environmental influences and contamination. They require regular maintenance and cleaning. The UHF method enables partial discharge detection within the Ultra High Frequency range (300 MHz to 3 GHz) [7]. This method offers great convenience as it allows measurements to be conducted at a certain distance from the transformer using a mobile UHF testing system [8]. UHF technology has the advantage of non-contact testing of electrical equipment without affecting its normal operation [9]. The UHF method is characterized by its high detection frequency and ease of installation, which has led to its increasing application in electrical equipment testing [10]. Therefore, this paper adopts the UHF method to detect and collect partial discharge signals from transformers.

In practical environments, the collected partial discharge signals are often mixed with noise due to complex electromagnetic conditions. In such environments, detecting and analyzing PD signals accurately is challenging. Therefore, effectively suppressing noise interference is a key challenge in the detection and analysis of partial discharge signals [11]. The main methods include filtering, Fast Fourier Transform (FFT) [12], wavelet transform [13], Empirical Mode Decomposition (EMD) [14], and Singular Value Decomposition (SVD) [15]. Filtering methods result in significant energy loss when directly removing irrelevant signals. FFT is a global noise analysis method that achieves good denoising effects for signals that change slowly over time [16]. Wavelet transform has strong time-frequency analysis capabilities but faces challenges in selecting wavelet bases and decomposition levels. Different wavelet bases and decomposition levels directly impact the denoising effect. The EMD algorithm can adaptively decompose signals, but it may encounter mode mixing when the modal frequencies are close, as well as over-decomposition and endpoint effects [17]. SVD can remove white noise from PD signals [18]. However, when the original signal contains multiple components, it is difficult for SVD to distinguish other components apart from white noise [19].

The Variational Mode Decomposition (VMD) algorithm is a novel adaptive and completely non-recursive method for mode estimation, proposed by Konstantin Dragomiretskiy in 2014 [20]. The VMD algorithm decomposes a signal into multiple mode components, each with different frequencies and amplitudes, to extract the signal’s time-frequency information. The VMD algorithm exhibits strong noise robustness, overcoming the issues associated with EMD and wavelet transforms mentioned above. However, the improper selection of parameters directly affects the performance and accuracy of the VMD algorithm in signal decomposition. In the literature [21], VMD parameters are determined based on empirical experience. However, relying solely on empirical experience introduces subjectivity and lacks objective evaluation, making it difficult to determine the accuracy of the decomposition. Heuristic optimization algorithms can address the parameter setting issue. In the literature [22], the Gray Wolf Optimization (GWO) algorithm is used to optimize VMD parameters, achieving good results. However, GWO has disadvantages such as slow convergence speed and weak global search capability [23]. The Whale Optimization Algorithm (WOA) has a simple principle and requires fewer parameter settings. In terms of function optimization, it outperforms the GWO in terms of convergence speed and solution accuracy [24]. Based on references [21,22,23,24], for a more accurate and efficient determination of VMD parameters to achieve the decomposition and denoising of PD signals, the adaptive optimization of VMD should be implemented. Even after denoising with VMD, residual noise may persist. The traditional techniques to suppress PD signal noise can be realized in the time domain (to identify certain repetitive noise) or in the frequency domain (using Fast Fourier Transform (FFT) to extract PD signals when PD and noise exhibit distinct frequency characteristics) [25]. However, FFT has inherent drawbacks such as spectral leakage, which limits its practical application and leads to the loss of time-domain information when processing signals in the frequency domain [26]. The wavelet threshold method can achieve both the time and frequency localization of signals, demonstrating excellent time-frequency analysis capabilities and wide applications in noise reduction within power systems [27]. Adaptive filtering techniques play a crucial role in various fields [28]. Hariri et al. used the Least Mean Squares (LMS) algorithm to remove noise from PD signals [29]. However, the LMS method requires prior knowledge of the expected signal, which is impractical for denoising real-world signals. The Normalized Least Mean Squares (NLMS) is an improvement over the LMS algorithm, as it does not require prior knowledge of the desired signal’s characteristics or the statistical properties of the effective signal and noise [30]. It automatically adjusts the filter coefficients by adjusting the difference between the input and reference signals to achieve optimal denoising objectives [31].

In response to the issue of noise interference in partial discharge (PD) signals, and building upon the advantages of the methods mentioned above, we put forward a method called Whale VMD combined with adaptive filtering and wavelet thresholding (WVNW). The WOA algorithm is employed to optimize the best combination of penalty factor (α) and decomposition levels (K). Founded on the kurtosis criterion, the noise constituents are separated, and the PD signal is reconstructed for initial denoising. The NLMS algorithm is then utilized to further reduce noise and smooth the PD signal by removing the interference caused by narrowband noise. Finally, the wavelet thresholding algorithm is applied to denoise the residual noise, achieving the denoising of the local discharge signal. Through simulation and experimental tests, compared with previous methods, the VMD-WT and EMD-WT methods, the results demonstrate that this method effectively suppresses noise interference while preserving more PD signal characteristics. The main contributions of this paper are as follows:

1. Introducing an adaptive VMD algorithm for the initial denoising of PD signals through decomposition and selection.
2. Leveraging the periodic nature of narrowband interference, incorporating the NLMS algorithm to further denoise the PD signals, and achieving signal smoothing. Additionally, utilizing the wavelet thresholding algorithm to effectively denoise residual white noise in the local discharge signals.
3. Experimental results demonstrate that, in comparison to existing methods, the proposed WVNW method effectively suppresses noise interference and better preserves the quantity and characteristics of PD signals.

## 2. Basic Theory

### 2.1. VMD Decomposition Principle

The Variational Mode Decomposition (VMD) algorithm is an adaptive non-recursive signal decomposition and time-frequency distribution estimation method [32]. This method extracts signals by solving a variational problem. The specific process is as follows.

First, we establish a variational problem. Given the original signal S, it is decomposed into *K* components *μ*. The objective is to ensure that each component has a finite bandwidth centered around a specific frequency while minimizing the sum of all bandwidths. We impose the constraint that the sum of all modes is equal to the original signal. The corresponding constrained variational expression is as follows [33]:

(1) $$\{\begin{array}{l} \underset{\left\{u_{k}\right\},\left\{\omega_{k}\right\}}{\min}\left\{\sum_{k=1}^{K}{\left\|\partial_{t}\left[\left(\delta (t)+\frac{j}{\pi t}\right)\cdot u_{k}(t)\right]e^{-j\omega_{k}t}\right\|}_{2}^{2}\right\}\\ \;s.t.\sum_{k=1}^{K}u_{k}(t)=f(t) \end{array}$$

In Equation (1), *u_k_*(*t*) represents the individual modes. By applying the Hilbert transform to *u_k_*(*t*), we obtain the analytic signal $\left(\delta (t)+\frac{j}{\pi t}\right)\cdot u_{k}(t)$. The central frequency $e^{-j\omega_{k}t}$ is estimated for the analytic signal $\left(\delta (t)+\frac{j}{\pi t}\right)\cdot u_{k}(t)$, and it is shifted from the spectral domain to the baseband. *f*(*t*) represents the input signal, *ω_k_* represents the center frequency corresponding to each mode, and δ(t) is the Dirac delta distribution function. Combining this information, we calculate the squared *L^2^* norm to estimate the bandwidth of each mode component.

Next, two parameters *α* and *λ* are introduced as penalty parameters and Lagrange penalty operators. Based on this, an unconstrained formulation is constructed, expressed as follows:

(2) $$L\left(\left\{u_{k}\right\},\left\{\omega_{k}\right\},\lambda \right)=\alpha \sum_{k=1}^{K}{\left\|\partial_{t}\left[\left(\delta (t)+\frac{j}{\pi t}\right)\cdot u_{k}(t)\right]e^{-jw_{k}t}\right\|}_{2}^{2}+{\left\|f(t)-\sum_{k=1}^{K}u_{k}(t)\right\|}_{2}^{2}+\langle \lambda (t),f(t)-\sum_{k=1}^{K}u_{k}(t)\rangle$$

Finally, the alternating direction method of multipliers (ADMM) is utilized to solve the saddle point of Equation (2).

(3) $${\hat{u}}_{k}^{n+1}(\omega )=\frac{\hat{f}(\omega )-\sum_{i<k}{\hat{u}}_{i}^{n+1}(\omega )-\sum_{i>k}{\hat{u}}_{i}^{n}(\omega )+\frac{{\hat{\lambda }}^{n}(\omega )}{2}}{1+2\alpha {\left(\omega -\omega_{k}^{n}\right)}^{2}}$$

(4) $$\omega_{k}^{n+1}=\frac{\int_{0}^{\infty }\omega {\left|{\hat{u}}_{k}^{n+1}(\omega )\right|}^{2}d\omega }{\int_{0}^{\infty }{\left|{\hat{u}}_{k}^{n+1}(\omega )\right|}^{2}d\omega }$$

(5) $${\hat{\lambda }}^{n+1}(\omega )={\hat{\lambda }}^{n}(\omega )+\tau \left[\hat{f}(\omega )-\sum_{k=1}^{K}{\hat{u}}_{k}^{n+1}(\omega )\right]$$

Equations (3)–(5) represent the updated formulas for *u_k_*, *ω_k_,* and *λ*. Initialize the parameters *u_1_, ω_1_*, *λ*, and n. Set up a loop process where n is incremented by 1. Update *u_k_*, *ω_k_*, and *λ* based on Equations (3)–(5). Set a predetermined convergence criterion *ε*, and check the condition based on Equation (6). When the condition is satisfied, stop the loop. By applying the inverse Fourier transform, obtain the modes {*u_k_*} and center frequencies {*ω_k_*} that satisfy Equation (1).

(6) $$\sum_{k=1}^{K}\frac{{\left\|{\hat{u}}_{k}^{n+1}(\omega )-{\hat{u}}_{k}^{n}(\omega )\right\|}_{2}^{2}}{{\left\|{\hat{u}}_{k}^{n}(\omega )\right\|}_{2}^{2}}<\varepsilon$$

### 2.2. WOA Algorithm

The Whale Optimization Algorithm (WOA) is a novel population-based optimization algorithm proposed by Mirjalili et al. from Griffith University, Australia in 2016 [34]. This algorithm is inspired by the hunting behavior of humpback whales. It consists of three stages: encircling prey, bubble-net hunting, and searching for prey (exploration) [35].

#### 2.2.1. Encircling Prey

The Humpback whale updates its position by surrounding its prey, which can be represented using the following model:

(7) $$H=\left|BZ^{*}(t)-Z(t)\right|$$

(8) $$Z(t+1)=Z^{*}(t)-A\cdot H$$

In the equation, *A* and *B* are coefficient constants, *t* represents the current iteration count, *Z*(*t*) represents the current position of the whale, and Z*(*t*) represents the best position. *H* represents the distance between the whale and the prey [36]. The coefficients *A* and *B* can be represented as follows:

(9) $$A=2a\cdot r_{0}-a$$

(10) $$B=2\cdot r_{1}$$

(11) $$a=2-\frac{2t}{T_{\max}}$$

In the equation, *r*_0_ and *r*_1_ are random numbers in the range [0, 1]. a is a value that decreases linearly from 2 to 0. *T_max_* represents the maximum number of iterations.

#### 2.2.2. Bubble-Net Hunting

When humpback whales are feeding, they spiral and release bubbles to create a bubble net. Their hunting behavior consists of two mechanisms: encircling prey and bubble-net hunting. The encircling prey mechanism can be represented by Equation (8). In bubble-net hunting, the position update between the whale and the prey is expressed using a logarithmic spiral equation. The corresponding mathematical model is as follows:

(12) $$Z\left(t+1\right)=H^{\prime }\cdot e^{bl}\cdot \cos\left(2\pi l\right)+Z^{*}\left(t\right)$$

(13) $$H^{\prime }=\left|Z^{*}(t)-Z(t)\right|$$

In the equation, *b* is a constant coefficient. *l* is a random number generated from the interval [−1, 1]. There are two hunting behaviors. Based on the probability *p*, one hunting behavior is selected. The corresponding position update formulas are as follows:

(14) $$Z(t+1)=\{\begin{array}{l} Z^{*}(t)-A\cdot H\;\;\;\;\;\;\;p<0.5\\ Z^{\prime }\cdot e^{bl}\cdot \cos(2\pi l)+Z^{*}(t)\;\;p⩾0.5 \end{array}$$

#### 2.2.3. Searching for Prey

The whale can update its position based on the distances to other individuals, thereby enhancing its global search capability and achieving the goal of random search. This approach helps achieve random searching. When |A| ≥ 1, a search individual randomly selects a whale for position update. The corresponding model is as follows:

(15) $$H_{\mathrm{rand}}=\left|B\cdot Z_{\mathrm{rand}}(t)-Z(t)\right|$$

(16) $$Z(t+1)=Z_{\mathrm{rand}}-A\cdot H_{\mathrm{rand}}$$

In the equation, $Z_{\mathrm{rand}}(t)$ and $H_{\mathrm{rand}}$ represent the position of the random whale and its distance from the prey, respectively.

### 2.3. Adaptive Filtering

**Figure 1:**
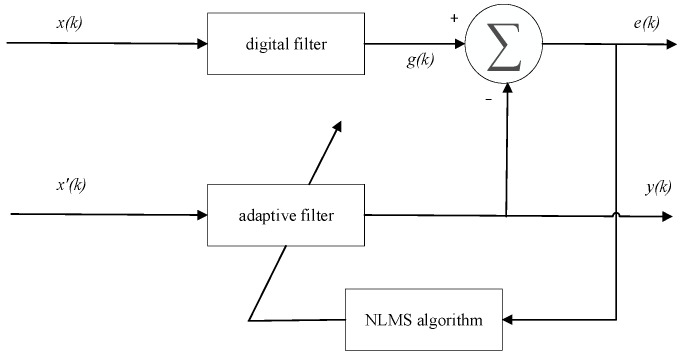
Adaptive filter principle diagram.

The LMS adaptive filter is capable of extracting useful signals from strong background noise. The NLMS algorithm, an extension of the LMS algorithm, is widely used in adaptive filtering [37,38]. NLMS is a variable step-size adaptive filtering algorithm that improves the convergence speed and accuracy compared to the fixed step-size of LMS. Its principle is shown in Figure 1 as follows:

*x*(*k*) is the input signal, which includes both noise and desired signal components. It is passed through a digital filter to obtain *g*(*k*). The difference between *g*(*k*) and the reference signal *x*′(*k*) results in the error signal *e*(*k*). The NLMS algorithm is then used to adjust the filter parameters to minimize the value of *e*(*k*). Based on the adjusted parameters, the input signal *x*(*k*) is filtered to obtain the desired output. 

### 2.4. Wavelet Thresholding Denoising 

**Figure 2:**
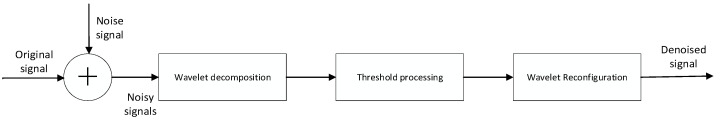
Wavelet thresholding denoising process flowchart.

Wavelet thresholding denoising is a denoising method introduced by Donoho et al. [39]. The denoising process can be divided into three main steps, as depicted in Figure 2:

(1). Decomposition: The target signal is decomposed using a chosen wavelet basis into N levels of wavelet coefficients.
(2). Thresholding: Each level of the decomposed wavelet coefficients is processed by applying an appropriate thresholding technique to obtain estimated wavelet coefficients, thereby achieving the denoising objective.
(3). Reconstruction: The denoised signal is reconstructed by performing an inverse wavelet transform using the wavelet coefficients.

### 2.5. Kurtosis Criterion

Kurtosis (*K*) is a statistical measure used to quantify the degree of peakedness or sharpness of a data distribution. It is calculated using the following formula:

(17) $$Ku=\frac{E\left[{\left(x-\mu \right)}^{4}\right]}{{\left(E\left[{\left(x-\mu \right)}^{2}\right]\right)}^{2}}$$

In the equation, *μ* represents the mean of the signal and *x* denotes the discrete signal. For partial discharge (PD) signals, they typically exhibit characteristics such as short duration, sharp rise, and sudden changes. PD signals tend to have larger peak values compared to noise. In terms of kurtosis, the kurtosis value for a signal without any occurrence of partial discharge is approximately equal to 3. However, when partial discharge events are present in the signal, the kurtosis value is significantly higher than 3 [40,41].

## 3. Partial Discharge Denoising Based on WVNW Method

### 3.1. Parameter Optimization of VMD Using WOA Algorithm

According to the information from the VMD theory, the exactness of the VMD algorithm is directly influenced by the two parameters K and *α*. However, the setting of these parameters is usually carried out manually, which introduces uncertainty and randomness, and requires a significant amount of time and effort to find the optimal values. To address this issue, this study utilizes the WOA approach for parameter optimization K and *α*. The WOA algorithm is known for its fast convergence and powerful global search capabilities. By employing the WOA algorithm, it is possible to efficiently and accurately obtain the optimal parameter combination.

The envelope entropy can indicate the sparsity of the signal characteristics. For each component obtained through VMD decomposition, if there is less noise and more valid PD signals, the value is larger. Conversely, the value is smaller. Therefore, the local minimum envelope entropy is chosen as the adaptation function for the WOA algorithm. The formula is as follows:

(18) $$\{\begin{array}{c} E_{p}=-\sum_{i=1}^{N}p_{i}\mathrm{lg}p_{i}\\ p_{i}=a(i)/\sum_{i=1}^{N}a_{i} \end{array}$$

In the equation, *N* represents the number of sampled points in the signal. *Ep* represents the envelope entropy. It is calculated based on the envelope signals *a*(*i*), which are obtained by performing the Hilbert transform on each IMF component of the signal. 

**Figure 3:**
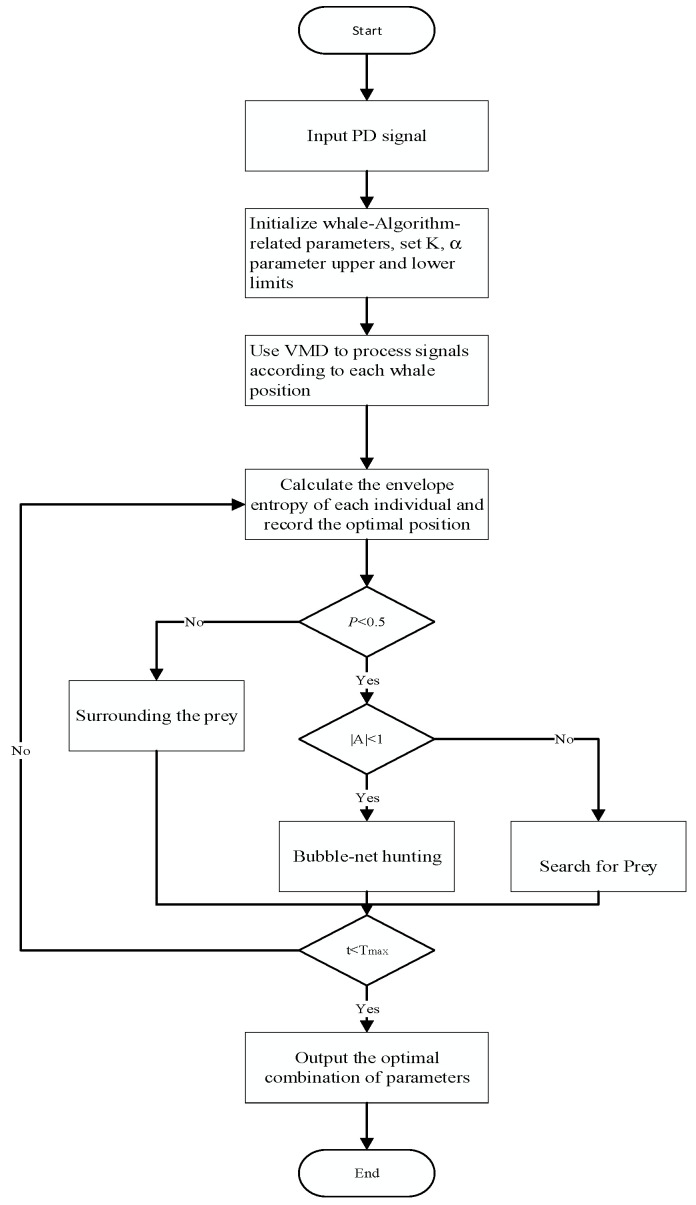
Flowchart of WOA optimization for VMD parameters.

The optimization of parameters K and *α* using WOA is illustrated in Figure 3. It involves a total of six steps, which are as follows:

(1) Initialize the WOA population and parameters (search dimension, population size, maximum iteration count). Set the range of K and *α* parameters and define the fitness function.
(2) Using the VMD algorithm, decompose the original signal based on the parameter range and calculate the fitness value for each parameter combination according to Equation (18).
(3) Utilize the optimization mechanism of the WOA algorithm to update the positions of individuals continuously. Compare the fitness values corresponding to each individual’s position and update the minimum fitness value.
(4) Iterate through steps 2 and 3 until the maximum iteration count, as initially set, is reached. In each iteration, update the positions of individuals and calculate the fitness value for the new positions.
(5) Output the optimal parameters K and *α*.
(6) Perform VMD decomposition using the optimal parameter combination to obtain the decomposed modal components.

### 3.2. Denoising Process for Partial Discharge Signals

This paper employs the WVNW method for denoising local partial discharge (PD) signals. First, the WOA method is employed to optimize the values of parameters K and *α*. Then, the parameter values are determined, and the PD signal is decomposed into components with different frequencies using the VMD algorithm. Subsequently, utilizing the kurtosis properties of the PD signal, components containing PD signal information are selected, and the signal is reconstructed using these components, achieving initial denoising. Finally, the NLMS algorithm and wavelet thresholding algorithm are utilized to further denoise the signal, resulting in a denoised PD signal. The detailed procedure is outlined as follows:

Step 1: Define the fitness function of the WOA algorithm as the local minimum envelope entropy and optimize the VMD parameters to obtain the optimal parameter combination.

Step 2: Based on the obtained K and A from Step 1, perform VMD decomposition on the noisy PD signal to obtain the modal components (IMFs).

Step 3: Calculate the kurtosis values of each modal component using the kurtosis criterion. Keep the modal components with kurtosis values greater than 3, while removing the ones with values less than 3 as they do not contain PD information. Reconstruct the PD signal to achieve initial denoising.

Step 4: Utilize the NLMS method to further denoise the signal and remove narrowband interference, resulting in a smoother waveform.

Step 5: Apply the wavelet thresholding method to perform the final denoising and obtain the denoised PD signal.

## 4. Simulation Analysis of Transformer Partial Discharge Signals

### 4.1. Simulation Model for PD Signals

The local partial discharge signals detected in the field are mostly attenuated oscillatory pulse signals, which can be represented by equivalent models such as the single exponential decay oscillation model (Equation (20)) and the double exponential decay oscillation model (Equation (21)) [42].

(19) $$s_{1}(t)=Ae^{-t/\tau }\sin2\pi f_{c}t$$

(20) $$s_{2}(t)=A\left(e^{-1.3t/\tau }-e^{-2.2t/\tau }\right)\sin2\pi f_{c}t$$

In the equation, *A* represents the signal amplitude, *τ* corresponds to the decay constant, and *f_c_* corresponds to the decay oscillation frequency.

According to the oscillation model described above, four partial discharge signals will be simulated with a sampling frequency of 20 MHz. The specific parameters for each PD signal are provided in Table 1. PD signals 1 and 3 follow the double exponential oscillation model, while PD signals 2 and 4 follow the single exponential oscillation model.

**Figure 4:**
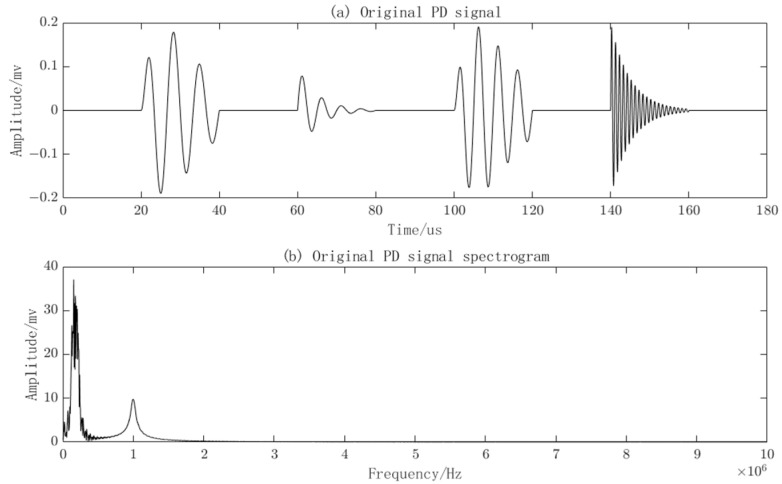
Original PD signals and their frequency spectra.

The clean PD signal and its corresponding frequency spectrum can be plotted based on the four PD signal models and their parameters in the table, as shown in Figure 4.

The local partial discharge signals detected in the field are mixed with narrowband interference and white noise. To simulate more realistic local discharge signals, the aforementioned two types of noise are added to the original PD signal. The white noise follows a Gaussian distribution N(0, 0.05^2^). The narrowband interference noise follows the following mathematical formula:

(21) $$f(t)=\sum_{i=1}^{5}0.02\sin\left(2\pi f_{i}t\right)$$

**Figure 5:**
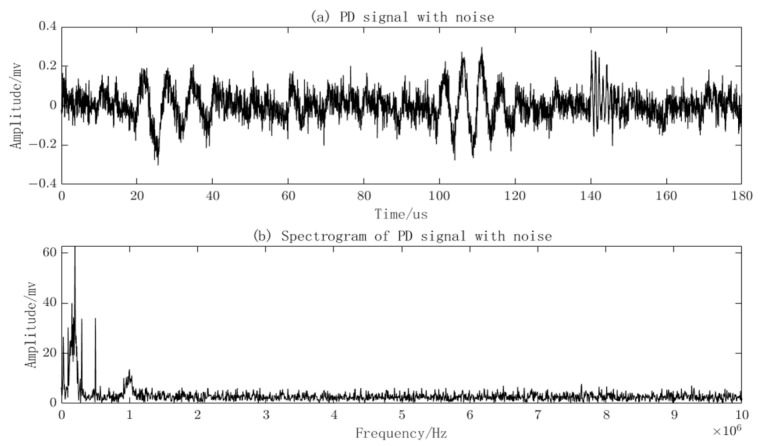
PD Signals with added noise and frequency spectra.

The amplitude of the narrowband interference is set to 0.02, and the frequencies *f_i_* are, respectively, set as: 0.03 MHz, 0.1 MHz, 0.2 MHz, 0.3 MHz, and 0.5 MHz. The PD signal with two types of noise added and its frequency spectrum is shown in Figure 5. The amplitude of the PD signal has changed after the addition of noise, and the noise is distributed throughout the waveform.

### 4.2. Simulating Denoising of Partial Discharge Signals

**Figure 6:**
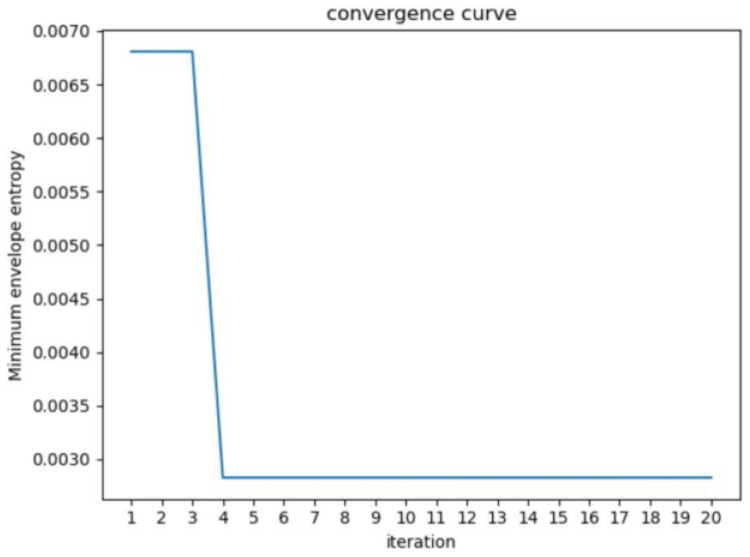
Local envelop entropy values of WOA-optimized VMD parameters for each generation.

The reasonable selection of parameters K and *α* is essential for the precise decomposition of PD signals using VMD. The WOA algorithm parameters are set with a population size of 100, a search dimension of 2, and a maximum iteration count of 20. The fitness function used is the local minimum envelope entropy. The VMD parameter optimization is demonstrated in Figure 6. It can be observed that the minimum value is reached in the fourth iteration, resulting in the optimal parameter combination of K and *α* as [7, 2807]. The WOA algorithm demonstrates fast convergence in optimizing parameters, outperforming manually set parameters. This reduces human effort, shortens the time required, and improves accuracy.

**Figure 7:**
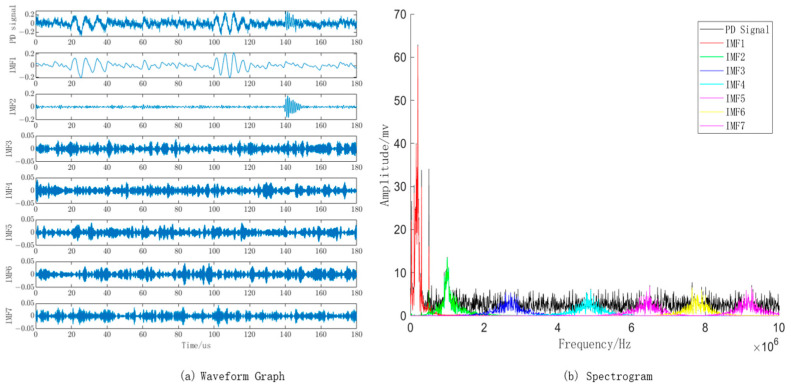
VMD decomposition of individual mode components and their corresponding spectral plots.

Based on the optimized parameter combination obtained from the WOA algorithm, the VMD algorithm is set with a mode number K of 7 and a quadratic penalty term *α* of 2807. The waveform and spectrogram of the PD signal with noise decomposed into IMF components by VMD are shown in Figure 7a,b, respectively. From the spectrogram, it is evident that the frequency spectrum of the IMF1 component closely matches the pristine PD signal with noise, and the highest point of the IMF2 component aligns with the PD signal around 1 MHz. This indicates that the parameter combination obtained through the WOA optimization algorithm is reasonable and offers a reliable decomposition of the PD signal with noise using VMD.

**Figure 8:**
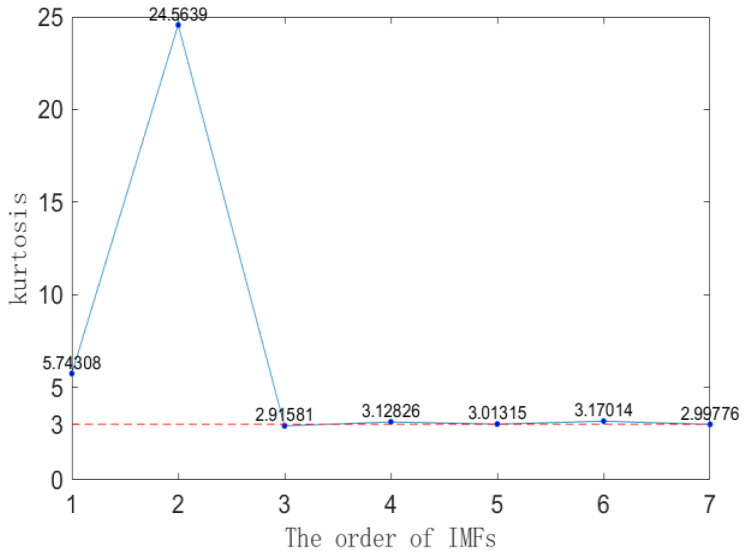
Kurtosis values of each mode component.

After setting the values of K and *α*, the PD signal contaminated with noise is decomposed into various IMF components through VMD. The kurtosis values of each IMF component are calculated as shown in Figure 8. According to the kurtosis criterion, IMF and IMF2 exhibit kurtosis values significantly greater than 3, indicating that they are the dominant components of the signal. IMF3 and IMF7 have kurtosis values below 3, suggesting that they represent noise components. Although the kurtosis values of IMF4-6 are larger than three, they are also close to three. Directly removing them would result in the loss of some valid signal information, so they need to be retained. The various IMF components, excluding the noise component, are reconstructed to accomplish the initial denoising process.

**Figure 9:**
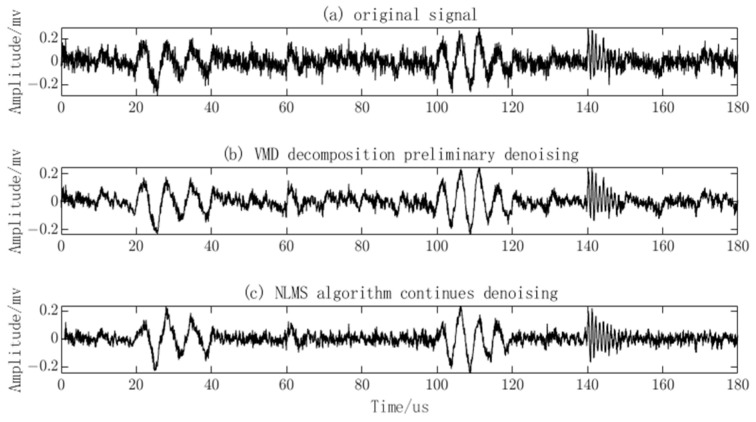
Denoising of partial discharge (PD) signals using VMD and NLMS.

The NLMS algorithm is applied to further denoise the reconstructed signal. The input signal is the PD signal after the preliminary denoising using the VMD algorithm. Due to the periodic nature of narrowband interference signals, they exhibit certain correlations at different time instants. Therefore, a time-division method is used to obtain a reference signal. The PD signal after denoising using the NLMS algorithm is shown in Figure 9. It can be observed that, after the preliminary denoising using the VMD algorithm, the noise has been weakened, but the waveform of the signal still exhibits significant fluctuations, and the PD signal is still submerged in the noise. However, after further denoising using the NLMS algorithm, it is evident that the noise has been effectively attenuated, and the waveform has become smoother. The distinctive features and quantity of the meaningful PD signal are now discernible.

**Figure 10:**
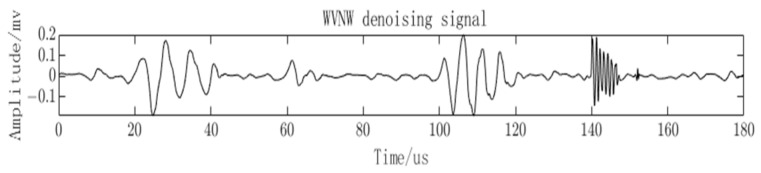
Denoising results of the proposed method for PD signals.

After denoising with VMD and NLMS algorithms, the noise has been effectively suppressed, but there is still residual white noise. To perform the final denoising, the wavelet thresholding algorithm is applied. The PD signal after denoising using the WVNW methodology proposed in this paper is visualized in Figure 10. It can be observed that the quantity and characteristics of the partial discharge signals have been restored.

### 4.3. Analysis of PD Signal Denoising Results

**Figure 11:**
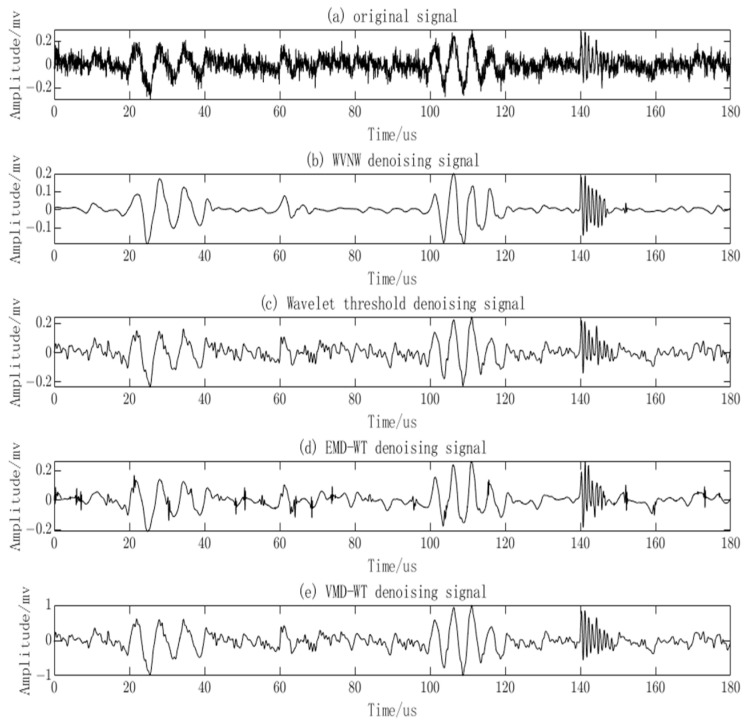
Comparison of PD signal denoising among four methods.

In this study, the WVNW method is employed for denoising the noisy PD signals. To examine the denoising capability of the proposed method, it is compared with the EMD-WT algorithm [43], the VMD-WT algorithm [44], and the Wavelet Thresholding approach, demonstrated in Figure 11. The EMD-WT algorithm removes a significant portion of the noise but also eliminates valid PD signals. The VMD-WT algorithm retains valid PD signals, but the noise removal is not thorough. The Wavelet algorithm removes some noise but also eliminates valid PD signals. In contrast, the proposed method achieves remarkable denoising results, with the recovery of valid PD signal quantity and characteristics. The transient features of the PD signals are also conserved. The denoised PD signals obtained using this method are more suitable for subsequent signal analysis.

The denoising capabilities of each method can be visually observed from the denoised waveform plots. To quantitatively evaluate the denoising effects of the methods, this study compared them using three evaluation metrics: root mean square error (RMSE), signal-to-noise ratio (SNR), and waveform similarity coefficient (NCC) [45]. The formulas are as follows:

(22) $$SNR=10*\log_{10}(\frac{\sum_{i=1}^{N}{\left|x(t)\right|}^{2}}{\sum_{i=1}^{N}{\left|x(t)-y(t)\right|}^{2}})$$

(23) $$RMSE=\sqrt{\frac{1}{N}\sum_{i=1}^{N}{\left|x(t)-y(t)\right|}^{2}}$$

(24) $$NCC=\frac{\sum_{i=1}^{N}x(t)*y(t)}{\sqrt{\left(\sum_{i=1}^{N}x^{2}(t)\right)*\left(\sum_{i=1}^{N}y^{2}(t)\right)}}$$

RMSE can measure the degree of signal distortion, with smaller values indicating less distortion. SNR can evaluate the denoising effect, with larger values indicating better performance. NCC assesses the similarity of signal waveforms, with values closer to 1 indicating a closer resemblance between the denoised and clean signals.

The evaluation results of the four methods are shown in Table 2. The WVNW method has a higher SNR than the other three methods, indicating better denoising performance. The NCC is closer to 1, indicating that the denoised signal is closer to the clean signal. The WVNW method also has the lowest RMSE, indicating a lower level of signal distortion and distortion rate. Based on these three evaluation metrics, the WVNW method outperforms the other methods in terms of denoising capability and the preservation of important PD signal features.

## 5. Analysis of Measured PD Signals

The PD signals detected by the substation mainly include white noise, random pulse interference, and periodic narrowband interference signals [46]. Pulse interference is easily eliminated due to its high-intensity and low-frequency characteristics. Local discharge signals are more seriously affected by white noise and periodic narrowband interference [47]. White noise mainly originates from random noise in communication lines, while narrowband periodic interference comes from radio communication, high-frequency protection, carrier communication, and higher-order harmonics, among others. In recent years, numerous researchers have conducted studies on denoising PD signals.

**Figure 12:**
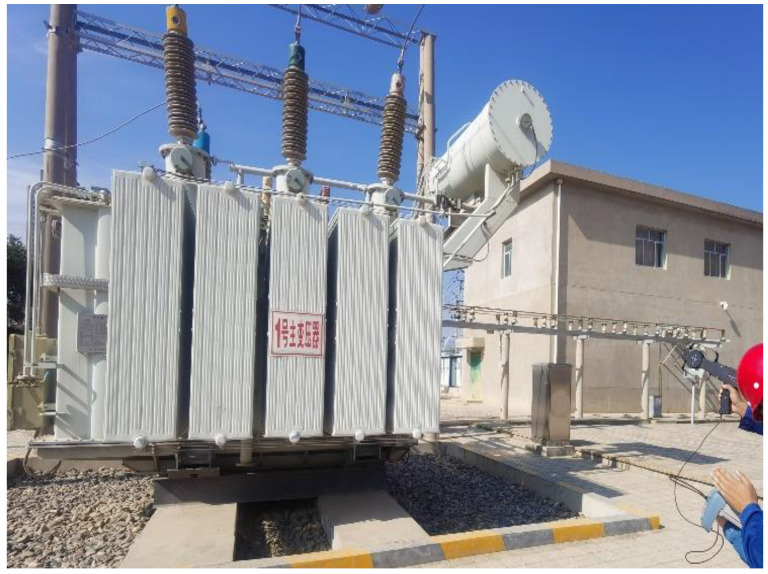
Field-collected PD signals.

**Figure 13:**
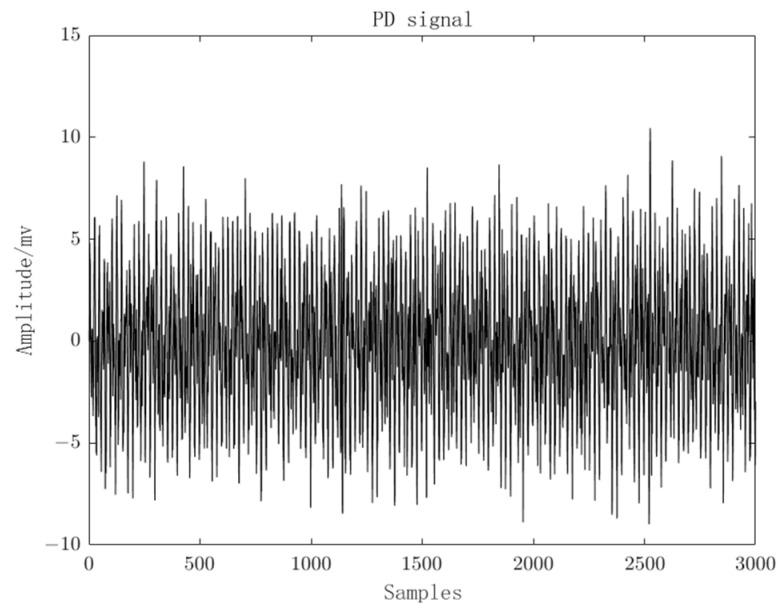
Measured PD signals.

To test the performance of the proposed method in this paper, field experiments were conducted at a 110 KV substation under the ownership of the State Grid Corporation of China. The transformer in this substation is a three-phase, 50 Hz transformer with a capacity of 31,500 KVA. The substation has been in operation for 25 years and is currently in the insulation degradation phase, exhibiting clear signs of partial discharge. The substation is depicted in Figure 12. For our experiments, a basic acquisition system was established using an Ultra High Frequency (UHF) sensor, a 3900 A receiver, and a laptop computer. This system was used to collect local discharge signals from the transformer. Subsequently, the collected PD signals were processed using Matlab, and the measured PD signals are shown in Figure 13. It can be observed that the PD signals are overwhelmed by noise, rendering them unidentifiable.

**Figure 14:**
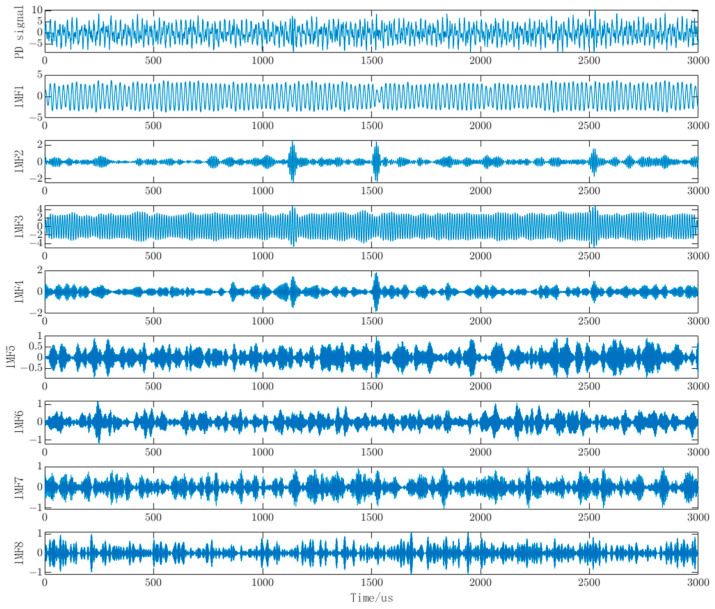
Individual modal components of the measured PD signal.

The VMD decomposition of the measured signal, as shown in Figure 14, reveals distinct characteristics. It is evident from the figure that IMF1 and IMF3 exhibit significant periodic oscillations, indicating that these two components are primarily dominated by narrowband interference. IMF2 and IMF4 display three distinct bursts of pulses, suggesting that they are dominated by PD signal components. The remaining components demonstrate random noise characteristics, implying that they are predominantly influenced by white noise. Given the substation’s location in a non-suburban area, surrounded by residential buildings and other structures, combined with the insights from the literature and the decomposition chart, it can be inferred that this measured PD signal is primarily affected by narrowband interference and white noise.

**Figure 15:**
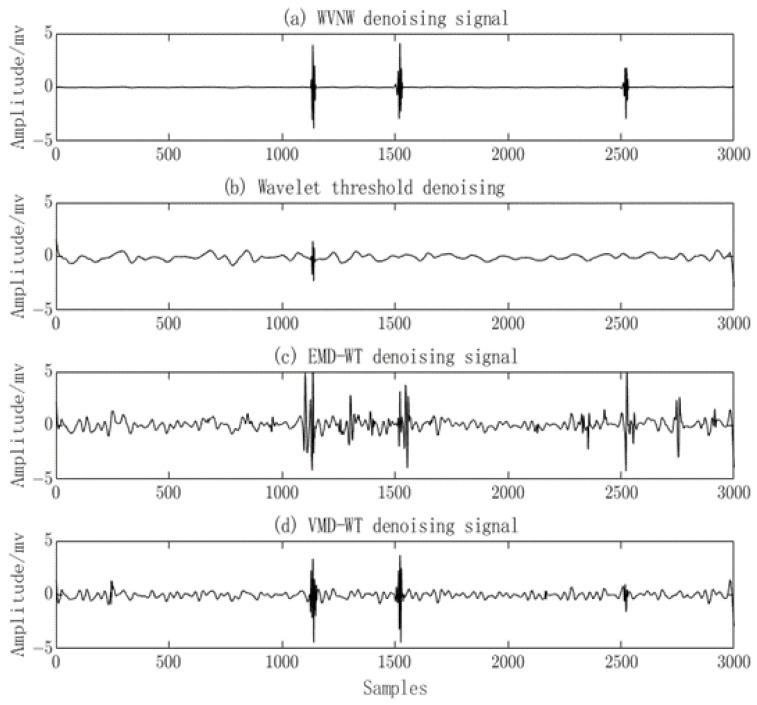
Comparison of denoising results for measured PD signals using four methods.

By using the proposed method, the EMD-WT algorithm, VMD-WT algorithm, and Wavelet Threshold algorithm, the measured signals were denoised as shown in Figure 15. It can be observed that the Wavelet Threshold algorithm effectively removes the noise but also eliminates valid PD signals, leaving only one valid PD signal remaining. The EMD-WT algorithm retains valid PD signals but the noise removal is incomplete, resulting in noticeable oscillations. The VMD-WT algorithm achieves a good denoising effect but fails to identify low-amplitude valid PD signals and still exhibits oscillatory behavior. In contrast, the proposed algorithm successfully suppresses noise without significant oscillations and effectively identifies low-amplitude PD signals.

Since the measured signal lacks information about the original ‘clean’ signal, it is not possible to calculate SNR, RMSE, and NCC. To quantitatively evaluate the denoising performance of the algorithms on the measured signal, the Noise Reduction Ratio (NRR) is introduced, which is calculated using the following formula [48]:

(25) $$NRR=10\cdot \left(\log_{10}\sigma_{1}^{2}-\log_{10}\sigma_{2}^{2}\right)$$

In the equation, $\sigma_{1}^{2}$ and $\sigma_{2}^{2}$ represent the standard deviations of the PD signal before and after denoising, respectively [49]. NRR is used to evaluate the prominence of the denoised signal, where a higher NRR indicates the better denoising performance of the algorithm. The NRR values for each algorithm can be found in Table 3. Among the four algorithms, the proposed algorithm in this paper achieves the highest NRR, indicating its superior denoising performance and effective preservation of the PD signal.

## 6. Conclusions

In this paper, a denoising method for transformer partial discharge is proposed, which is based on the Whale VMD algorithm combined with adaptive filtering and wavelet thresholding(WVNW). It effectively suppresses the interference of noise on PD signals. Through simulation and experimental analysis of PD signal denoising, the main results and conclusions obtained from this study are as follows:

(1) The VMD algorithm can decompose the local discharge signals into mode components with different frequencies, effectively preserving the waveform characteristics of the local discharge signals. The WOA, with the objective of local minimum envelope entropy, can efficiently optimize the parameters. The complementary nature of these two methods enables the accurate decomposition of the PD signals.
(2) The selected mode components after VMD decomposition are further filtered and reconstructed based on the kurtosis criterion, achieving initial denoising. The Adaptive Filter, implemented with the NLMS algorithm, is applied to further denoise the PD signals by removing narrowband interference noise and smoothing the PD signals. The remaining white noise is then eliminated using Wavelet Thresholding.
(3) By denoising simulated PD signals and PD signals measured at transformer stations, and comparing them with traditional methods, EMD-WT, and VMD-WT methods, the findings suggest that the method proposed in this paper is more effective in preserving the waveform characteristics. It effectively suppresses noise and preserves more PD signals and their features.

## Figures and Tables

**Table 1:** Simulated parameters of PD signals.

Pulse Model	A/mv	*τ*/us	*f_c_*/Mhz
1	1	10	0.15
2	0.1	5	0.2
3	1	10	0.2
4	0.2	5	1

**Table 2:** Values of the three evaluation metrics.

Denoising Method	RMSE	SNR	NCC
Wavelet Threshold	0.17866	3.3741	0.81987
EMD-WT	0.15737	4.4765	0.83948
VMD-WT	0.14279	5.3207	0.85405
WVNW	0.082764	9.6404	0.94542

**Table 3:** NRR evaluation indicator values.

Denoising Method	NRR
Wavelet Threshold	0.4812
EMD-WT	1.2208
VMD-WT	2.7272
WVNW	3.6701

## Data Availability

Not applicable.
